# Drinking and Night-Time Driving May Increase the Risk of Severe Health Outcomes: A 5-Year Retrospective Study of Traffic Injuries among International Travelers at a University Hospital Emergency Center in Thailand

**DOI:** 10.3390/ijerph18189823

**Published:** 2021-09-17

**Authors:** Vorapot Sapsirisavat, Wiriya Mahikul

**Affiliations:** 1 Department of Preventive Medicine and Family Medicine, Faculty of Medicine, Burapha University, Chonburi 20131, Thailand; vorapot.sa@go.buu.ac.th; 2 Faculty of Medicine and Public Health, HRH Princess Chulabhorn College of Medical Science, Chulabhorn Royal Academy, Bangkok 10210, Thailand

**Keywords:** traffic accident, road traffic injury, severe health outcomes, multinomial logistic regression analysis, international travelers, Thailand

## Abstract

Road traffic injury (RTI) is a leading cause of death in developing countries. This burden affects not only locals, but also international travelers. Data on international travelers with RTIs in Thailand, especially from a medical perspective, are limited. This study aimed to analyze the factors associated with severe health outcomes following RTIs among international travelers at a university hospital emergency center in Thailand from January 2015 to December 2019. The retrieved data consisted of demographics, risks, preventive factors, and health outcomes. The severity of outcome was classified as fatality, hospitalization, or non-severe. A multinomial logistic regression model was used to identify the possible determinants of severity of health outcome among international travelers with RTI. A total of 720 travelers with RTIs (69% males; 82.5% were Southeast Asian) were included, with a mean age of 28.5 years. Of these, 144 (20%) had severe health outcomes: 64 (9%) fatalities and 80 (11%) hospitalizations. The level of severity of outcome was not associated with travelers’ demographics, but was associated with conventional risk factors, i.e., motorcycle use, alcohol/drug use, night-time driving, and less use of seatbelt/helmet. In a multinomial logistic regression analysis, alcohol drinking (adjusted odds ratio (AOR) 2.53, 95% confidence interval (CI) 1.41–4.55) and night-time driving (AOR 2.54, 95% CI 1.36–4.75) were associated with hospitalization. Patients who had a history of tetanus vaccination were less likely to die (AOR 0.37, 95% CI 0.17–0.81). In conclusion, one-fifth of RTIs resulted in severe health outcomes, and 9% were fatal. Road safety campaigns in Thailand should target travelers of all nationalities. Interventions that enhance travelers’ safety practices and proper preparation for road accidents should be explored further.

## 1. Introduction

Road traffic injury (RTI) is estimated by the World Health Organization (WHO) to be the leading cause of death for people aged less than 30 years and the eighth-leading cause of death for people of all ages [1]. Annually, motor vehicle crashes lead to approximately 1.3 million fatalities and an additional 20–50 million injuries worldwide. Ninety percent of road traffic deaths occur in developing countries, although they hold only 54% of the world’s registered vehicles [2,3]. During 2013–2016, the rates of road traffic death were highest in Africa (26.6/100,000 population) and Southeast Asia (20.7/100,000). Thailand ranks among the countries with the highest rate of traffic deaths in the region and globally: 32.7/100,000 population [1]. RTIs are not only problematic for local populations; this was the leading cause of death among international travelers in developing countries [4,5]. In high-risk settings, the proportion of traffic injury is more notable in travelers compared with the local population [6]. In Jeju Island, Korea, transportation-related injuries often occur among travelers more than local residents [7]. According to data from the Thai Road Safety Collaboration (TRSC), during 2019–2021, there were at least 12,754 RTIs and 907 deaths among international travelers in Thailand (fatality rate 907/12,745 = 7.1%). The fatality rate was much higher than among resident Thais who had RTIs during the same period (39,794/2,423,544 = 1.6%) [8].

Risk factors of severe health outcome following RTIs comprise road users (host), vehicles (agent), and the road (including other environmental factors such as location and pre-hospital factors). Male drivers, alcohol/drug use, exceeding the speed limit, under-maintained vehicles, night-time driving, and poor road and traffic management are factors increasing the severity of an event. Using personal restraints (seatbelt/helmet), safer vehicles and improved road hazard treatment are preventive measures during the event; first aid, rescuer skills, and accessibility of rescue teams are preventive measures post-event [9,10,11,12,13,14,15,16,17,18,19]. While factors influencing the severity outcome were well-established in previous research, most of the literature only focused on local road users. The epidemiological pattern of RTIs and the associated consequences can be varied among different world regions [17]. Travelers from different countries may be less familiar with road safety practices and emergency medical response systems at the destination, especially in a high-risk setting, like Thailand [6]. TRSC data from 2016–2020 showed that more than half of the deceased travelers following RTIs were Asian (67%) followed by European (13%) and African (10%). The top three countries of origin were Myanmar, China, and Laos [8].

Although one-third of RTI events are preventable, pre-travel preparation often fails to take these precautions. A survey of travel medicine clinics worldwide reported that 70% discuss personal safety during pre-travel consultations, while almost all emphasize infection prevention [20,21]. Although medical certificate is required for the driver license application in Thailand, discussion on RTIs’ prevention is not a mandatory and mostly missed during a medical visit. Road safety campaigns targeting international travelers have also been insufficient; not only is there a language barrier [22], but there are also cultural differences, particularly handedness of road traffic between countries [23]. Although international travelers have been affected greatly by RTIs in Thailand, studies on road traffic accidents among this group are limited [24,25,26]. In shortness of research data and attention, it is important to characterize the travelers and the risk factors associated with severe health outcome following RTIs. A better understanding of the risk factors associated with severe health outcomes following RTIs among international travelers will highlight and support policy-makers to consider additional preventive measures. Therefore, the primary objective of this research is to identify the associated factors that increase the severity of health outcome among international travelers with RTIs in Thailand.

## 2. Materials and Methods

### 2.1. Overall Study Design and Samples

We conducted a retrospective study using medical records of international patients who had a traffic injury resulting in a visit to the Burapha University Hospital emergency center during the period January 2015–December 2019. This hospital in Chonburi province is affiliated with the Faculty of Medicine Burapha University, the only medical school in eastern Thailand. The hospital emergency center is mainly responsible for RTIs occurring in the Saensuk sub-district and nearby Chonburi province. The road characteristic can be classified mainly as minor arterials in the context of a suburban area [27]. This area is one of the areas with the most international travelers—not only migrant workers, but also expatriates, tourists, and other travelers. We defined all international patients as travelers to include all these categories. Chonburi is one of the areas in Thialand where the majority of deaths from RTIs among travelers occurred [8].

### 2.2. Data Measurement

Data from the medical record included travelers’ demographics, risk factors, and preventive measures. The demographics data included gender, age, nationality, and handedness of road traffic in their home countries. The risk data consisted of host (alcohol/illicit drug use), agent (motorized 2–3 wheelers vs. other types of vehicles), and environment (night-time driving); the preventive measures were use of restraints (helmet, seatbelt) and history of tetanus vaccination. The outcome (dependent variable) was level of severity of the road traffic accident (fatality, hospitalization, or non-severe). A fatal injury was defined if travelers died immediately or after visiting the emergency center due to RTIs. Patients who died at the scene were not included as they were not transported to the emergency center. Hospitalization was defined if the traveler survived, but the RTI was severe enough to require hospital admission. Otherwise, travelers were classified into the non-severe group. There were no repeated RTI events for any one individual during 2015–2019, thus each traveler was only categorized once.

### 2.3. Data Analysis

A total of 720 RTI cases were collected from all traffic injuries recorded from 2015 to 2019 at the Burapha University Hospital emergency center. Categorical variables were summarized using frequencies and percentages, while continuous variables were analyzed and summarized with the mean value. A chi-square test was performed to assess the association for categorical data between factors and severity outcomes (fatality, hospitalization, and non-severe), while the association with continuous data was assessed by analysis of variance (ANOVA) test. All tests were two-sided, and *p*-values < 0.05 were considered statistically significant.

Univariate and multinomial logistic regression analysis were performed at a 5% level of significance. The associations between severity outcomes and risk factors were analyzed using a multinomial logistic regression model. The model was used to predict the relationship between demographic characteristics, potential risk factors, potential preventive factors, and the severity of health outcome. The multinomial logistic model is specified as

(1) Y=βX+ε

where Y is severity outcomes (fatality, hospitalization, and non-severe), β is the vector of coefficient estimates, X is the vector of parameters, and ε is an independently and identically distributed generalized extreme value error term. The exponentiated values of the estimated coefficient ($e^{\beta }$) referred to the odds ratio (OR). It can be used to explore how variables affect the choice of reference outcome (non-severe) compared with another outcome. The increase or decrease in odds ratio refers to the risk or protective factor of a specific injury severity level relative to the reference category, respectively [28].

Descriptive statistics and multinomial logistic regression analyses were performed using SPSS version 23.0 software (IBM, Armonk, NY, USA). The likelihood ratio test was used to evaluate the significance of the estimated model by comparing the estimated model to the null (model without any independent variables) [29]. The result of the goodness-of-fit test was used to examine the quality of the model when the deviance *p*-values are greater than 0.05 [30]. Moreover, the values of pseudo-R^2^ indicate a reasonable level of fit.

## 3. Results

### 3.1. Description of Overall Travelers with RTIs

From a total of 720 RTI cases, the annual incidence of about 150 cases was consistent over the 5 years of the study period, with a mean age of 28.5 years. More than half of the injured cases were male or were aged less than 35 years, at 68.9% and 78.5%, respectively. Five hundred and ninety-four (82.5%) of the injury cases came from Southeast Asia, followed by Europe (7%). The road user types among cases were most commonly drivers of motorized 2–3 wheelers (79.2%), followed by pedestrians (6.1%), cyclists (5.6%), and car occupants (4.3%). The possible risk factors among injured cases were reported as follows: 13.2% drank alcohol, 27.9% did not use a seatbelt/helmet, and 58.6% were driving at night. Only one-fifth had a history of tetanus vaccination. Of the 720 injured travelers, 144 (20%) had severe health outcomes (9% died and 11% were hospitalized).

### 3.2. Factor Associated with Severe Health Outcome

Table 1 displays statistically significant differences by Pearson’s chi-square and ANOVA tests in the level of severity following RTIs by demographic characteristics, potential risk factors, and potential preventive factors. The majority of severe outcomes occurred among motorcycle riders (68.8% of fatal cases, 83.8% of hospitalized cases) and night-time drivers (65.6% of fatal cases, 77.5% of hospitalized cases). However, travelers’ demographics and different handedness of road traffic between their home country and Thailand were not associated with outcome severity. Among potential risk factors, motorized 2–3 wheelers, alcohol/drug use, and night-time driving were each significantly associated with outcome severity (*p*-values of 0.034, <0.01, and <0.01, respectively). For the preventive factors, using of seatbelt/helmet and history of tetanus vaccination were associated with a lower level of severity outcome (both *p*-values < 0.01).

### 3.3. Multinomial Logistic Model on the Severity Outcome

The forward selection method was employed in the multinomial logistic regression analysis. The score test was used to determine the variable entry at a significance level of 0.05, while removal testing based on the maximum partial likelihood estimates at a significance level of 0.1 [31]. Finally, only three factors remained (as shown in Table 2). The likelihood ratio test shows that all of them are statistically significant at a 0.05 level of significance, and the overall prediction accuracy is desirable (79.1%) compared with previous literature [31,32].

The crude odds ratio (OR) and adjusted odds ratio (AOR) were the predicted outcomes of the multinomial logistic regression model without and with adjusted the confounding factors, respectively. The non-severe outcome is a reference category and two other outcomes were defined as the comparator: (1) hospitalization versus non-severe and (2) fatality versus non-severe.

The resulting univariate multinomial logistic regression model is presented in Table 3. The odds of being hospitalized after RTIs in those who drank alcohol and drove at night as compared with non-severe cases were 2.94 times greater (95% CI = 1.66–5.20) and 2.77 times greater (95% CI = 1.60–4.81) than those who did not drink alcohol and did not drive at night, respectively. In addition, the odds of hospitalization and death after RTIs among those who had a history of tetanus vaccination as compared with non-severe cases were 2.22 and 2.78 times lower, respectively, than those who did not (95% CI = 1.12–4.35 and 95% CI = 1.28–5.88, respectively).

As shown in Table 4, after adjustment for travelers’ potential risk factors by multivariate analysis with multinomial logistic regression, the odds of being hospitalized after RTIs in those who drank alcohol and drove at night as compared with non-severe cases were 2.53 times greater (95% CI = 1.41–4.55) and 2.54 times greater (95% CI = 1.36–4.75) than those who did not drink alcohol and did not drive at night, respectively. In addition, the odds of death after RTIs among those who had a history of tetanus vaccination as compared with non-severe cases were 2.70 times lower than those who did not (95% CI = 1.23–5.88).

## 4. Discussion

We describe 720 international travelers who had RTIs and visited the Burapha University Hospital emergency center during 2015–2019. Demographics of injured travelers were identical to those collected in the national database; travelers from Southeast Asia and Europe were the groups who had the highest number of RTI incidents in Thailand [8]. The majority of injured travelers, following the global trend, were younger males and motorcycle riders [15,33,34]. One-fifth of the travelers with traffic injury experienced severe health outcomes. The fatality rate of 9% was comparable to the national data on foreigners who had RTIs in Thailand and much higher than local Thai residents [8].

The severity of the outcome was significantly associated with conventional risk factors, i.e., drinking alcohol and night-time driving [17,35,36,37]. The study supports that the conventional risk factors for severe traffic accidents are universal, contributing both locals and travelers. Besides human and environmental factors, the problem is also prominent specifically where road traffic laws have not been strictly implemented. The WHO reported that Thailand had underperformed on 4 of 12 global road safety performance targets; most of these were related to insufficient law enforcement [38]. However, during the worst of the coronavirus 2019 (COVID-19) situation, there was enhanced law enforcement to restrict alcohol sales and enforce the night-time curfew in Thailand; overall, reported traffic accidents decreased by 60.8%. Traffic-related injuries and deaths were reduced by 63.4% and 56.7%, respectively, during this period [39].

Although international travelers’ demographics were not associated with the severity of outcomes, the high fatality rate in this group implies that travelers themselves possess additional risk factors. A study among foreigners with RTIs in Chiang Mai, Thailand found the time delay between RTIs and incident report to be a significant problem [40]. Besides the possible language barrier, foreigners may lack a practical understanding of what to do after a road traffic crash [40]. Delays in notification and receiving care for those involved in RTIs increase the severity of outcomes [12,17,19,41]. Our study found lower fatalities among travelers who had a tetanus vaccination; history of a tetanus vaccination may represent preparedness and receiving care either from a previous injury or pre-travel preparation [42]. Although there was no repeated traffic injury among our subjects during the study period, travelers may have previously encountered a traffic accident or had other pervious injuries, and may have thus received an education on injury prevention along with the tetanus vaccination. They may have thus been better equipped with practical knowledge on whom to contact and where to go when an injury occurs.

There are limitations to our study. First, the use of retrospective data restricted us from clarifying some data collected when travelers with RTIs presented at the emergency center; for instance, use of illicit drugs could not be totally ruled out or confirmed given the potential alteration in a patient’s consciousness. Therefore, the possibility of illicit drug use was not included in the regression analysis. In addition, we could not retrospectively collect some potentially related factors such as road and vehicle conditions, pre-travel preparation, duration of stay in Thailand, and the reason for tetanus vaccination. Travelers who died at the scene were also missed. Second, the collected data from this single center were strongly weighted toward travelers from Southeast Asia. The results, hence, may not be generalizable to every population of travelers, although travelers’ demographics from our data were in general accordance with the national data, reflecting the current situation across Thailand. Third, the fatality rate may have been overestimated because our study retrieved the data from the hospital emergency center; some travelers with slight injuries may not have visited a hospital. Lastly, this study used retrospective data and a cross-sectional study design, preventing us from making causal inferences between factors and the level of outcome severity.

Our study presented hospital-based 5-year retrospective data among travelers with an RTI who visited an emergency center. The high fatality rate might influence transportation and public health authorities. The policy should be more focused on these vulnerable road users, especially in areas with significant numbers of foreigners, i.e., Bangkok, Chiang Mai, Phuket, and Chonburi. On an individual level, when travelers visit a hospital for a medical certificate as a part of a driver’s license application, the doctor might assess if travelers are aware that the risk of RTIs is significantly higher compared with in their countries of origin; the doctor can discuss preventive measures and preparation for the occurrence of an RTI, including pre-exposure tetanus vaccination. In term of public health, a road safety campaign such as a public awareness campaign of the risks of drinking alcohol and preventing night-time driving accidents should be included for international travelers who might have a higher risk of severe health outcome.

## 5. Conclusions

Among the hospital database of 720 international travelers with RTIs, we report that one-fifth of traffic injuries resulted in severe health outcomes (including a 9% fatality rate). The level of outcome severity was associated with conventional risk factors, including alcohol drinking and night-time driving. Outcome severity did not significantly vary by travelers’ demographics, although we suspect that accessibility to post-crash care may be a potential factor influencing the high fatality rate. Travelers’ preparations and experiences, represented by tetanus vaccination history, may protect travelers from more severe health outcomes. Traffic injury is a universal problem, but the rate of RTI fatalities among travelers in Thailand is significantly higher than the rate in travelers’ home countries and the rate among Thai people. As international travelers are essential for economic growth in Thailand, stakeholders should increase efforts and campaigns to promote road safety and improve post-crash care, focusing on this population with a high fatality rate. Further research agendas should prospectively compare the factors influencing severity of injuries both crash and post-crash. The comparison with local residents at the same geodemographic should be made. The interventional study on individual travelers and public health measures can further support policy maker to curb the incidence and severity of RTIs among international travelers in Thailand.

## Figures and Tables

**Table 1:** Comparison of the characteristics of international travelers with traffic injuries at the Burapha University Hospital emergency center, classified by severity of health outcome (*n* = 720).

	Outcome (*n* = 720)			*p*-Value
	Non-Severe *n* = 576 (%)	Hospitalization *n* = 80 (%)	Fatality *n* = 64 (%)	
Demographic characteristics				
Male sex	388 (67.4)	59 (73.8)	49 (76.6)	0.195
Age, mean years (range)	28.39 (1–80)	27.01 (1–74)	31.22 (9–80)	0.316
Southeast Asian	475 (82.5)	70 (87.5)	49 (76.6)	0.229
Right-hand traffic	531 (92.2)	74 (92.5)	58 (90.6)	0.955
Risks				
Motorized 2–3 wheelers	459 (79.8)	67 (83.8)	44 (68.8)	**0.034**
Alcohol drinking	65 (12.7)	24 (33.8)	6 (9.4)	**<0.01**
Possibility of illicit drug use	445 (77.3)	62 (77.5)	61 (95.3)	**0.013**
Night-time driving	318 (55.4)	62 (77.5)	42 (65.6)	**<0.01**
Preventions				
Seatbelt/helmet use	46 (8.9)	2 (2.9)	1 (1.6)	**0.001**
History of tetanus vaccination	139 (24.1)	12 (15.0)	9 (14.1)	**0.004**

NOTE. Data are numbers (%) of patients, unless otherwise indicated. Chi-square and ANOVA tests were used for assessing the association between categorical variables and continuous variables, respectively. Significant differences are marked in bold.

**Table 2:** Likelihood ratio test.

Variable	−2 Log Likelihood of Reduced Model	Chi-Square	Degrees of Freedom	*p*-Value
Intercept	106.412	0	0	-
Alcohol drinking	135.737	30.325	4	<0.001
Night-time driving	116.595	10.184	2	0.006
History of tetanus vaccination	117.847	11.435	4	0.022

**Table 3:** Crude odds ratio (OR) for each factor comparing levels of outcome severity at the Burapha University Hospital emergency center.

General Characteristics		Hospitalization vs. Non-Severe			Fatality vs. Non-Severe	
	Estimate (*β*)	S.E.	OR (95% CI)	Estimate (*β*)	S.E.	OR (95% CI)
Risk						
Alcohol drinking	1.08	0.29	**2.94 (1.66–5.20)**	−0.61	0.45	0.54 (0.22–1.32)
Nighttime driving	1.02	2.81	**2.77 (1.60–4.81)**	0.43	0.28	1.54 (0.89–2.64)
Prevention						
History of tetanus vaccination	−0.43	0.26	**0.45 (0.23–0.89)**	−1.01	0.39	**0.36 (0.17–0.78)**

S.E. = standard error; OR = odds ratio by univariate analysis with multinomial logistic regression; significant odds ratios are marked in bold.

**Table 4:** Adjusted odds ratio (AOR) for each factor comparing levels of outcome severity at the Burapha University Hospital emergency center.

General Characteristics		Hospitalization vs. Non-Severe			Fatality vs. Non-Severe	
	Estimate (*β*)	S.E.	AOR (95% CI)	Estimate (*β*)	S.E.	AOR (95% CI)
Risk						
Alcohol drinking	0.93	0.30	**2.53 (1.41–4.55)**	−0.636	0.46	0.63 (0.23–1.81)
Night-time driving	0.93	0.32	**2.54 (1.36–4.75)**	0.332	0.29	1.28 (0.72–2.29)
Prevention						
History of tetanus vaccination	−0.69	0.38	0.49 (0.24–1.04)	−0.985	0.39	**0.37 (0.17–0.81)**

S.E. = standard error; AOR = adjusted odds ratio; adjusted for alcohol, driving time, and tetanus history by multivariate analysis with multinomial logistic regression; significant adjusted odds ratios are marked in bold. Note: Goodness-of-fit-statistics: deviance = 106.76; degrees of freedom = 10; *p* = 0.328. −2 log likelihood: the initial model only with the constant: 167.76; the final model: 106.76; pseudo-R^2^: Cox and Snell = 0.091; Nagelkerke = 0.124; McFadden = 0.072. Overall prediction accuracy: 79.1%.

## Data Availability

The data presented in this study are available on request from the corresponding author. The data are not publicly available due to ethical.
